# Higher Plasma *Myo*-Inositol in Pregnancy Associated with Reduced Postpartum Blood Loss: Secondary Analyses of the NiPPeR Trial

**DOI:** 10.3390/nu16132054

**Published:** 2024-06-27

**Authors:** Hsin F. Chang, Hannah E. J. Yong, Han Zhang, Jui-Tsung Wong, Sheila J. Barton, Philip Titcombe, Benjamin B. Albert, Sarah El-Heis, Heidi Nield, Judith Ong, Luca Lavelle, J. Manuel Ramos-Nieves, Jean-Philippe Godin, Irma Silva-Zolezzi, Wayne S. Cutfield, Keith M. Godfrey, Shiao-Yng Chan, the NiPPeR Study Group

**Affiliations:** 1 Department of Obstetrics and Gynaecology, National University Hospital, Singapore 119074, Singapore; hsin_fang_chang@nuhs.edu.sg (H.F.C.); judith_ong@nuhs.edu.sg (J.O.); 2 Singapore Institute for Clinical Sciences, Agency for Science, Technology and Research, Singapore 117609, Singapore; hannah_yong@sics.a-star.edu.sg (H.E.J.Y.); zhang_han@sics.a-star.edu.sg (H.Z.); ray_wong@sics.a-star.edu.sg (J.-T.W.); 3 MRC Lifecourse Epidemiology Centre, University of Southampton, Southampton SO16 6YD, UK; s.j.barton@soton.ac.uk (S.J.B.); pt@mrc.soton.ac.uk (P.T.); se@mrc.soton.ac.uk (S.E.-H.); h.nield@soton.ac.uk (H.N.);; 4 Liggins Institute and a Better Start—National Science Challenge, The University of Auckland, Auckland 1023, New Zealand; b.albert@auckland.ac.nz (B.B.A.); w.cutfield@auckland.ac.nz (W.S.C.); 5 NIHR Southampton Biomedical Research Centre, University of Southampton & University Hospital Southampton NHS Foundation Trust, Southampton SO16 6YD, UK; 6 Nestlé Research Centre, 1000 Lausanne, Switzerland; luca.lavalle1@rd.nestle.com (L.L.); manuel.ramos@rdls.nestle.com (J.M.R.-N.); jean-philippe.godin@rdls.nestle.com (J.-P.G.); 7 Research & Development, Nestlé Product Technology Center—Nutrition, 1800 Vevey, Switzerland; irma.silvazolezzi@nestle.com; 8 Department of Obstetrics & Gynaecology, Yong Loo Lin School of Medicine, National University of Singapore, Singapore 119228, Singapore

**Keywords:** postpartum hemorrhage, inositols, B-vitamins, vitamin D, prenatal nutritional supplement

## Abstract

We previously reported that a combined *myo*-inositol, probiotics, and enriched micronutrient supplement (intervention) taken preconception and in pregnancy reduced postpartum blood loss (PBL) and major postpartum hemorrhage compared with a standard micronutrient supplement (control), as secondary outcomes of the NiPPeR trial. This study aimed to identify the intervention components that may contribute to this effect. Associations of plasma concentrations of *myo*-inositol and vitamins B2, B6, B12, and D at preconception (before and after supplementation), early (~7-weeks), and late pregnancy (~28-weeks) with PBL were assessed by multiple linear regression, adjusting for site, ethnicity, preconception BMI, parity, and previous cesarean section. Amongst 583 women, a higher concentration of *myo*-inositol in early pregnancy was associated with a PBL reduction [β_adj_ −1.26 (95%CI −2.23, −0.29) mL per µmol/L *myo*-inositol increase, *p* = 0.011]. Applying this co-efficient to the increase in mean 7-week-*myo*-inositol concentration of 23.4 µmol/L with the intervention equated to a PBL reduction of 29.5 mL (~8.4% of mean PBL of 350 mL among controls), accounting for 84.3% of the previously reported intervention effect of 35 mL. None of the examined vitamins were associated with PBL. Therefore, *myo*-inositol may be a key intervention component mediating the PBL reduction. Further work is required to determine the mechanisms involved.

## 1. Introduction

Postpartum hemorrhage (PPH) remains a leading cause of maternal mortality and morbidity. World Health Organization (WHO) statistics suggest that 60% of maternal deaths in developing countries are attributable to PPH, resulting in more than 100,000 maternal deaths worldwide annually [1]. Uterine atony (impaired uterine contraction) after childbirth is the most common etiology [2]. Risk factors of uterine atony include prolonged labor, previous cesarean section, high parity, multiple pregnancies, and maternal obesity [3].

Although administration of uterotonic agents is an established universal practice to promote uterine contractility post-delivery, they are not completely protective of atonic PPH [4,5]. Thus, there is a need to find complementary approaches acceptable to pregnant women, as part of a road map set out by the World Health Organization [6], to more effectively combat against PPH. One such additional strategy is prenatal nutritional supplementation. There is a further advantage if these are also affordable and easy to assimilate into existing cultures and systems operating in low-to-middle-income settings.

Studies suggest that several micronutrients are involved in promoting blood coagulation and myometrial contractility, and may have a role in reducing PPH. For instance, deficiencies in vitamin D [7,8,9] and zinc [10,11] are associated with impaired uterine contractility, leading to prolonged labor and PPH in human and animal studies. Indeed, antenatal vitamin D supplementation reportedly reduced the risk of severe PPH [12]. However, these findings were inconsistent between trials, and meta-analyses of studies have not found a significant difference in PPH with either vitamin D [13] or zinc supplementation [14]. Meanwhile, supplementation of multiparous dairy cows with folate and vitamin B12 lowered the incidence of labor dystocia [15], suggestive of improved uterine contractility.

*Myo*-inositol, a carbohydrate naturally present in cells and abundant in fruits, grains, and nuts, is another promising nutritional intervention [16]. Inositol is a component of many signaling pathways (e.g., phosphoinositides, inositol phosphoglycans) [16] and is being trialed in pregnancy for the prevention and treatment of gestational diabetes [17,18]. Studies in pre-clinical models demonstrated that *myo*-inositol could improve myometrial contractility partly by regulating calcium fluxes [19].

The NiPPeR (Nutritional Intervention Preconception and during Pregnancy to maintain healthy glucosE metabolism and OffspRing health) multi-center double-blinded randomized controlled trial previously reported that, compared with a standard micronutrient supplement, the intervention of a combined *myo*-inositol, probiotics, and enriched micronutrient supplement reduced postpartum blood loss (PBL) by 35 mL (95% confidence intervals [CI] −70.0, −3.5; 10% of the mean PBL among controls) [20] and the incidence of major PPH (adjusted risk ratio 0.44, 95%CI 0.20, 0.94) as secondary outcomes of the trial [21]. The current study aimed to identify the component(s) in the NiPPeR intervention that mediated the reduction in PBL.

## 2. Materials and Methods

### 2.1. Study Design and Participants

Approval was obtained from research ethics committees at each study site: United Kingdom, Singapore, and New Zealand. All participants provided written informed consent. This trial was prospectively registered at ClinicalTrials.gov NCT02509988, and oversight and monitoring were provided by an independent data and safety monitoring committee.

This current study is a secondary analysis of data collected in the NiPPeR trial. We have previously published the trial protocol and reported no difference in the primary outcome of the level of gestational glycemia at 28 weeks’ gestation with intervention compared with the control [21]. Briefly, between 2015 to 2017, the study recruited 1729 women who were planning conception, aged 18–38 years, from the community. Exclusion criteria were pregnant/lactating, assisted conception (apart from taking clomiphene or letrozole alone), serious food allergy, diabetes mellitus, taking hormonal contraception, metformin, systemic steroids, anticonvulsants or treatment for HIV or Hepatitis B or C in the past month. Participants were electronically randomized in a 1:1 ratio to be given a control supplement containing folic acid, iron, calcium, iodine, and β-carotene or an intervention supplement that additionally included *myo*-inositol (4 g daily), vitamin B2, vitamin B6, vitamin B12, vitamin D, zinc, and probiotics (*Lactobacillus rhamnosus* and *Bifidobacterium animalis* sp. *Lactis*) [22]. Supplements were consumed twice daily from preconception following randomization until delivery. Over 96% of participants achieved the pre-specified threshold of good adherence (>60% supplement intake) from recruitment to delivery [21].

### 2.2. Data Collection

Data on maternal age, ethnicity, preconception smoking, household income, parity, and history of previous cesarean section were collected from participants upon recruitment by interviewer-administered questionnaires. Pre-pregnancy body mass index (BMI) was calculated from measured pre-pregnancy weight divided by measured height squared (kg/m^2^). Details on the mode of delivery, total estimated blood loss at delivery, and birthweight (kg) were extracted from medical records. Gestational age at birth (weeks) was derived from a previously published algorithm using a combination of the last menstrual period date, cycle length and regularity, and early pregnancy ultrasound scan [20]. Neonates were defined as large-for-gestational-age (LGA; birthweight > 90th percentile standardized for gestation and sex) and small-for-gestational-age (SGA; birthweight < 10th percentile) using RCPCH 2009 UK-WHO growth charts [23].

### 2.3. Laboratory Analyses

Plasma obtained at four time points: baseline and after randomization and supplementation commencement at preconception (mostly 23–30 days [range 21–42]; median 28 days [IQR 22, 31]), in early pregnancy (7-weeks; median 7.4 weeks [IQR 7.1, 7.9]), and in late pregnancy (28-weeks; median 27.7 weeks [IQR 27.2, 28.3]), were batch-analyzed. Plasma concentrations of vitamins B2 (riboflavin), B6 (pyridoxal-5-phosphate), and D (25-hydroxyvitamin D3) were analyzed by targeted methods of liquid chromatography with tandem mass spectrometry (LC-MS-MS) while plasma concentrations of vitamin B12 (cobalamin) were measured by a microbiological assay; all at Bevital Laboratory (Bergen, Norway), as previously described [24,25]. A recently developed and validated ultra-high-performance-liquid chromatography tandem mass spectrometry (UHPLC-MS/MS; Neotron, Italy, in collaboration with Nestlé Research, Switzerland) method was used to quantify plasma and baseline urinary *myo*-inositol and *scyllo*-inositol concentrations [26].

### 2.4. Statistical Analysis

Data distribution was assessed through histograms and Kolmogorov–Smirnov tests. All nutrient variables were log_e_-transformed to achieve approximate normality and standardized across the whole dataset to derive standard deviation (SD) scores before regression analysis. This enabled the strength of the association with blood loss to be compared among different nutrients as well as across collection time points. Estimated blood loss was also log_e_-transformed for regression analyses. The resulting regression coefficients (beta; 95%CI) represent the % change in mL blood loss per increase of one standard deviation (SD) of log_e_ nutrient concentration. For results of interest, these were then converted to mL per unit concentration of nutrient for reporting using the anti-log_e_ equivalent of mean blood loss of the entire cohort (347.23 mL) and the anti-log_e_ equivalent of one standard deviation of each nutrient. To derive an indicative estimate of blood loss (in mL), which could be ascribed to particular nutrients of interest, the coefficient (in mL per unit concentration of nutrient) was then applied to the mean increase in nutrient concentration with the NiPPeR intervention compared with the control (anti-log_e_ mean nutrient in intervention group minus anti-log_e_ mean nutrient in control group).

For examining each nutrient on a continuum, control and intervention data were analyzed as a combined group. Multiple linear regression modeling was conducted adjusting for (1) study site only (since estimations of blood loss are well-documented to vary between clinical settings) and (2) clinically important factors at recruitment that have prognostic influence on postpartum blood loss based on existing literature and that improved model-fit when assessed by stepwise forward regression: pre-pregnancy BMI (kg/m^2^), parity (nulliparous, parous), previous cesarean section, and age (year). Further adjustment for ethnicity and household income did not improve model fit. We then performed further analyses by mutually adjusting for the vitamins and *myo*-inositol components simultaneously within the same model. Potentially different nutrient-PBL associations between various groupings of parity, previous cesarean section, and across BMI, were also investigated, reporting any statistical interactions. To further confirm the identified relationships we conducted Pearson correlation analyses stratified by site, assessing the correlations between each nutrient of interest and residuals of pre-adjusted blood loss for preconception BMI and parity by linear regression.

Sensitivity analyses were performed to examine the robustness of associations. Firstly, we additionally adjusted for inherent variations in baseline inositol processing represented by inositol metabolism (plasma *scyllo*-inositol:*myo*-inositol ratio) and urinary excretion (urine:plasma *myo*-inositol ratio). Secondly, we excluded cases involving obstetric events known to be associated with increased blood loss, namely cesarean section delivery and large-for-gestational-age infants. These models additionally served as exploratory analyses to determine if these factors could potentially lie on the causal pathway.

There was no imputation for missing data. Results were considered statistically significant when the two-tailed probability was <0.05. Statistical analyses were carried out using Stata Software Release 15 (StataCorp, College Station, TX, USA).

## 3. Results

### 3.1. Participant Characteristics

Of the 1729 women recruited, 585 conceived and had a singleton live birth between April 2016 to January 2019 (Supplementary Figure S1). Of these, 583 (99.7%) provided peripartum data (290 control and 293 intervention) for this sub-study. The mean age of participants was 30.3 years with an average pre-pregnancy BMI of 23.7 kg/m^2^. The majority of participants were White Caucasian (59.2%), 79.8% never smoked, and 63.3% were nulliparous. At baseline, participants had a median plasma *myo*-inositol concentration of 21.9 µmol/L; 7.7%, 1.7%, 10.7%, and 41.9% had low status for vitamins B2, B6, B12, and D, respectively (Table 1).

### 3.2. Associations between Nutrients and Postpartum Blood Loss

**Figure 1:**
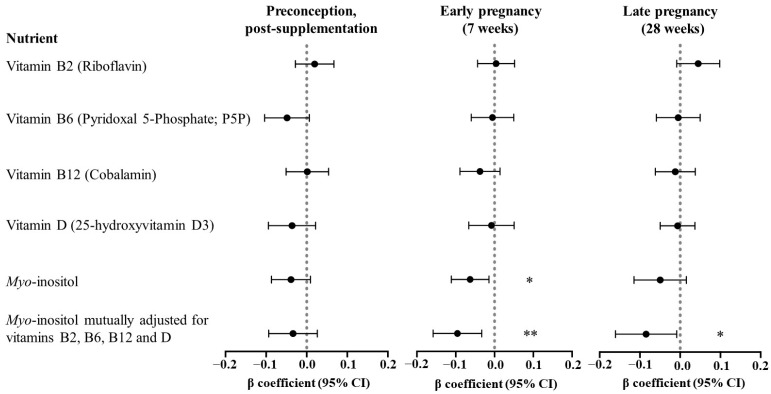
Associations of maternal plasma concentrations of vitamins and *myo*-inositol with postpartum blood loss. Plasma was collected at preconception post-supplementation (N = 581), 7 weeks of pregnancy (N = 579), and 28 weeks of pregnancy (N = 579). Log_e_-transformed and standardized; coefficients expressed as log_e_ mL blood loss per SD log_e_ increase of measured nutrient. Regression models were adjusted for site, preconception BMI, parity, previous cesarean section, and maternal age. Model fit (represented by R^2^): 7-week *myo*-inositol (adjusted: 0.206; mutually adjusted for other nutrients: 0.213) and 28-week *myo*-inositol (adjusted: 0.197; mutually adjusted for other nutrients: 0.207). Statistical significance: * *p* < 0.05, ** *p* < 0.01. Abbreviations: CI, confidence interval.

In analyses combining both intervention and control groups, adjusting for site only, higher concentrations of maternal preconception plasma *myo*-inositol and vitamin B6, and 7-week plasma *myo*-inositol and vitamin B12 were associated with reduced PBL (Supplementary Figure S2). However, following adjustment for the other covariates only maternal plasma *myo*-inositol remained associated with reduced PBL (Figure 1): 7-week *myo*-inositol [β_adj_ −1.26 (95%CI −2.23, −0.29) mL per µmol/L *myo*-inositol increase, *p* = 0.011], 28-week *myo*-inositol [β_adj_ −0.99 (−2.30, 0.31), *p* = 0.135]. There was a similar trend for *myo*-inositol measured at preconception post-supplementation [β_adj_ −0.24 (−0.50, 0.03), *p* = 0.078].

Taking the intervention supplement containing 4 g *myo*-inositol daily led to a 2-fold (23.4 µmol/L) increase in the mean 7-week *myo*-inositol concentration compared with control [26]. By applying the 7-week-*myo*-inositol-associated-PBL coefficient to this plasma concentration increase, a reduction in PBL by 29.5 mL could be predicted. This equates to 8.4% of the average PBL in the control group, which would account for approximately 84.3% of the earlier reported intervention effect of a decrease in PBL by 35 mL compared with the control [20]. The association between 7-week *myo*-inositol and reduced blood loss was similar in control and intervention groups (interaction term 7-week-*myo*-inositol*intervention-group, *p* = 0.839). In contrast, maternal plasma concentrations of vitamins B2, B6, B12, and D at all three time points were not associated with PBL in fully adjusted models.

When we accounted for concentrations of vitamins B2, B6, B12, and D by mutual adjustment in the same regression model as *myo*-inositol, the association between plasma *myo*-inositol concentrations at 7 weeks and at 28 weeks with a reduction in PBL became more apparent [7-week-*myo*-inositol: β_adj_ −1.92 (95%CI −3.17, −0.66) mL per µmol/L, *p* = 0.003; 28-week-*myo*-inositol: −1.64 (−3.26, −0.20), *p* = 0.048] (Figure 1).

**Figure 2:**
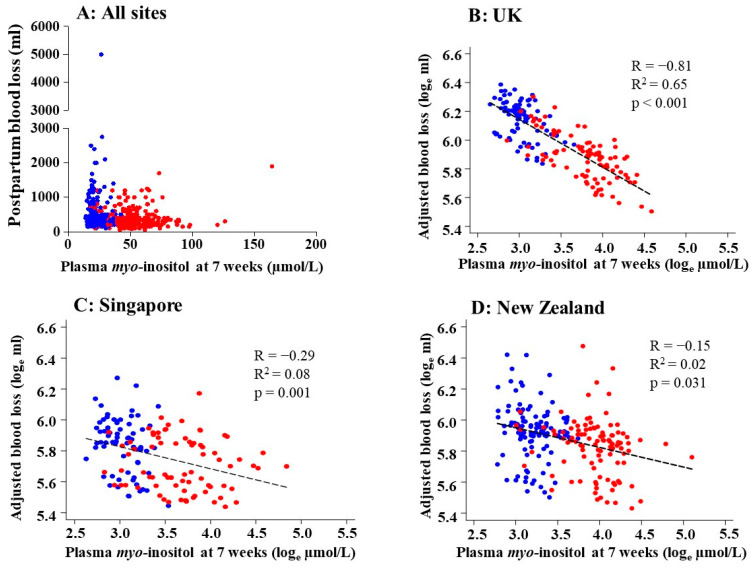
Correlations between the maternal plasma concentration of *myo*-inositol at 7 weeks of gestation and postpartum blood loss. (**A**) All sites combined showing unadjusted raw data and (**B**–**D**) site-stratified plots using log_e_-transformed plasma *myo*-inositol data and log_e_-transformed blood loss adjusted for preconception BMI and parity generated using linear regression. Pearson’s correlation (R), R-squared (R^2^), and *p*-values are shown. The participants in the NiPPeR control group are represented by blue dots while those in the NiPPeR intervention group (taking the supplement containing *myo*-inositol) are represented by red dots. UK: n = 177, Singapore: n = 146, New Zealand: n = 214.

There was a consistent inverse relationship between 7-week *myo*-inositol and blood loss observed at all three sites, with the strongest correlation observed among UK participants (R −0.81, R^2^ 0.65; *p* < 0.001) (Figure 2). Inverse associations between 7-week-*myo*-inositol and PBL were similarly observed across different groupings of parity (interaction-*p* = 0.63), previous cesarean section (interaction-*p* = 0.74), and preconception BMI (interaction-*p* = 0.80).

### 3.3. Sensitivity Analyses

**Figure 3:**
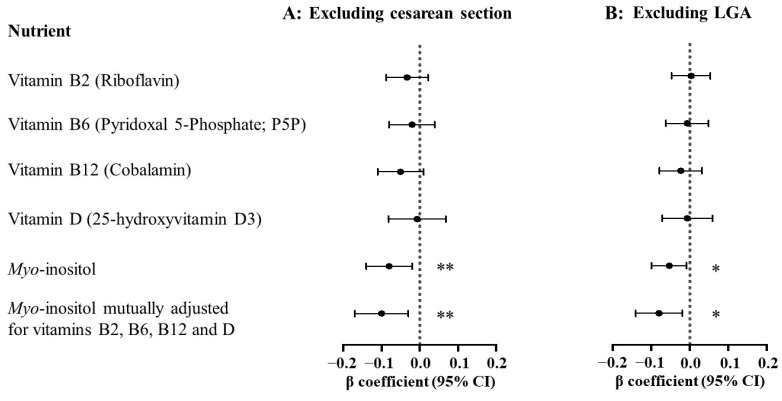
Sensitivity analyses examining associations between maternal plasma concentrations of vitamins and *myo*-inositol at 7 weeks of gestation with postpartum blood loss after excluding cases of (**A**) cesarean section delivery (n = 169 excluded), or (**B**) large-for-gestational-age neonate at birth (n = 43 excluded). Maternal plasma concentrations of vitamins and *myo*-inositol were log_e_-transformed and standardized while postpartum blood loss was log_e_-transformed. Coefficients expressed as % change in mL blood loss per standard deviation (SD) increase in log_e_ nutrient concentration. Regression models were adjusted for site, preconception BMI, parity, previous cesarean section, and maternal age. Statistical significance: * *p* < 0.05, ** *p* < 0.01. Abbreviations: CI, confidence interval; LGA, large-for-gestational-age.

The association between 7-week *myo*-inositol and PBL reduction was robust in sensitivity analyses. With additional adjustment for individual inherent variations in inositol metabolism and excretion as a potential confounder, 7-week *myo*-inositol remained associated with PBL reduction, with no change in results [β_adj_ −1.26 (95%CI −2.24, −0.28) mL per µmol/L, *p* = 0.012]. After excluding cases involving obstetric events that are known risk factors for increased PBL, 7-week-*myo*-inositol-PBL associations were not substantially altered either: excluding cesarean section delivery (n = 169 excluded) [β_adj_ −1.77 (−2.88, −0.66), *p* = 0.002] and LGA cases (n = 43 excluded) [β_adj_ −1.18 (−2.15, −0.20), *p* = 0.018] (Figure 3). Similar trends of association were also observed between 28-week *myo*-inositol and PBL. There remained no associations between plasma concentrations of vitamins B2, B6, B12, and D measured at any of the three time points and postpartum blood loss, following the exclusion of cesarean section and LGA cases.

## 4. Discussion

This secondary analysis of the NiPPeR study data suggests that *myo*-inositol was a key component of the NiPPeR combined nutritional supplement that contributed substantially to the observed reduction in PBL previously reported with the NiPPeR intervention when compared with controls [20,21]. Of note, a higher plasma *myo*-inositol concentration was associated with reduced PBL across a continuum, even below the 500 mL blood loss threshold defining PPH. Our analyses also indicate that achieving a relatively higher plasma *myo*-inositol concentration from early pregnancy onward may be an important consideration for reducing PBL. Meanwhile, none of the other vitamins present in the NiPPeR intervention were associated with PBL. The relationship between plasma *myo*-inositol and reduced PBL remained evident even among cases not complicated by known risk factors for PPH, such as cesarean section deliveries or LGA infants. This finding suggests that the mechanism of the effect of *myo*-inositol does not necessarily involve the modulation of these particular risk factors. To our knowledge, this is the first report of a link between a higher maternal plasma *myo*-inositol concentration during pregnancy and reduced PBL.

### 4.1. Comparisons with Published Literature

Other trials of *myo*-inositol supplementation in pregnancy did not report on PBL nor PPH outcomes, likely because the data were not collected as they were not pre-specified outcomes. Notably, our results are inconsistent with a vitamin D trial that reported a reduction in major PPH [12]. Our finding of a lack of vitamin D effect on blood loss is, however, concordant with a meta-analysis of vitamin D trials [13] and a more recent vitamin D publication [27]. Furthermore, we previously reported that the NiPPeR intervention supplement did improve the maternal status of vitamins B2, B6, B12, and D during pregnancy leading to a wider range of plasma vitamin concentrations across the cohort [25]; despite this, no associations with PBL were observed. The initially observed links between higher plasma concentrations of preconception (post-supplementation) vitamin B6 and early pregnancy vitamin B12 with PBL reduction in the site-only adjusted models were no longer evident in the fully adjusted models. This finding suggests the strong influence of confounding factors rather than a direct causal effect in these relationships. Such factors may include a lower BMI, which is associated with improved myometrial contractility, and a healthier diet rich in vegetables, fruits, and grains, which can concurrently increase vitamin B6, vitamin B12, and *myo*-inositol plasma concentrations.

### 4.2. Clinical Implications

The estimation of a *myo*-inositol effect of 29.5 mL reduction in blood loss (with the increase in plasma *myo*-inositol at 7 weeks’ gestation with a 4 g daily supplement) amounts to approximately 84.3% of the mean difference of 35 mL in blood loss between the NiPPeR intervention and control groups that we reported previously [20]. This suggests that *myo*-inositol may predominantly account for the intervention effect. The potential roles of other components of the NiPPeR supplement, including zinc and probiotics, in regulating postpartum blood loss remain to be assessed. Although the magnitude of *myo*-inositol-related blood loss reduction of ~8.4% may seem small, its clinical significance must be interpreted in the context of a nutritional supplement study rather than a pharmacological-based intervention. By comparison, the anti-fibrinolytic drug, tranexamic acid, which is being trialed at delivery for PPH prophylaxis, has been estimated to reduce PBL by ~20% in clinical trials [28]. Every element that may reduce blood loss post-delivery would add to the cumulative effect of a constellation of variables, which could have a considerable overall influence.

Despite the NiPPeR study participants being generally healthy women delivering in high-resource settings with advanced medical care and widespread use of prophylactic oxytocin against PPH, it is remarkable that variations in plasma *myo*-inositol in pregnancy could still be associated with a discernible difference in PBL. Moreover, imprecisions in PBL estimations in healthcare are well-recognized, as is variation in the methods used for such estimations across clinical settings [29]. Yet, this did not obscure our finding of associations with *myo*-inositol, which was consistent across study sites. Further studies in more diverse populations, including in low-resource settings, are needed to determine if *myo*-inositol supplementation would have a similar or even more potent effect.

### 4.3. Postulated Mechanisms of Effect

Given that *myo*-inositol is a central component of many key players involved in intra- and inter-cellular signaling pathways (e.g., phosphoinositides, inositol phosphates, inositol phosphoglycans), as well as a component of important regulators of plasma membrane function (e.g., phosphatidylinositols), increased *myo*-inositol bioavailability through supplementation could influence many biological processes. There are several potential mechanisms by which *myo*-inositol may reduce PBL. A study on myometrial tissue from non-pregnant rats showed that inositol can promote uterine contractility through the regulation of calcium influxes [19]. Indeed, within the NiPPeR trial, we observed that participants who received the *myo*-inositol-containing NiPPeR intervention had a shorter second stage of labor duration and reduced operative delivery risk for delayed second stage of labor progress [20], events that are hugely influenced by uterine contractility. Enhanced uterine contractility may also promote more efficient occlusion of the uterine blood vessels once the placenta is detached from the uterine wall and prevent excessive PBL through these vessels.

If the early pregnancy concentration of *myo*-inositol is indeed most influential on PBL, as suggested by our findings, then underlying mechanisms may also include effects on early placental development. Deep trophoblastic invasion into the maternal uterine wall and blood vessels takes place in the first half of gestation, with critical transformation of maternal spiral arteries, facilitating blood flow through the placental bed to ensure adequate maternal-fetal exchange [30]. This is a tightly regulated process involving multiple hormonal, inflammatory, and metabolic elements, thus, it is conceivable that the facilitation of *myo*-inositol-related second messenger signals may optimize this entire process, with later consequences on PBL. Another mechanism by which *myo*-inositol may reduce PBL could be through effects on the maternal coagulation system by priming the clotting function of platelets. Inositol can be derivatized to form inositol pyrophosphate, which is important for the generation of polyphosphates that are released by activated platelets to promote clotting by enhancing the clotting cascade and strengthening the fibrin clot structure [31,32].

### 4.4. Strengths and Weaknesses

An advance over previous studies is our longitudinal measures of maternal plasma *myo*-inositol, vitamins B2, B6, B12, and D at preconception, and in early and late pregnancy. This approach may allow the identification of the periods of greatest potential impact of nutrient levels on PBL. Nonetheless, our resolution remains limited since nutrients were not measured between 7 weeks’ and 28 weeks’ gestation, nor later in pregnancy, as such, we cannot more precisely define the optimal gestational window for the achievement of a high *myo*-inositol level. Moreover, the preconception collection took place between weeks and up to a year before pregnancy and the actual periconception levels may have differed from those measured at our preconception study visit time point. Further studies evaluating the optimal timing and duration for *myo*-inositol supplementation and defining the ideal concentration range to achieve across pregnancy in order to impact PBL and other pregnancy outcomes are needed to fully unravel the therapeutic potential of *myo*-inositol. For example, the potential benefit of early *myo*-inositol supplementation on PBL reduction needs to be weighed against the potential side-effect of a higher early pregnancy *myo*-inositol marginally increasing post-prandial glycemia later in gestation [26]. Additionally, this exploratory study, even though conducted within the context of a randomized controlled trial, reported on adjusted associations between plasma *myo*-inositol and PBL reduction, which may not have accounted for yet-to-be-discovered confounders. The study may, therefore, have over- or under-estimated the effect of *myo*-inositol. Mechanistic studies and a new clinical trial of prenatal *myo*-inositol supplementation with PBL as the primary outcome would be needed to establish causation. Evidence from such studies would inform whether public health policies should be implemented to promote higher dietary intakes of *myo*-inositol-rich foods or *myo*-inositol supplementation from early pregnancy for the purposes of postpartum blood loss reduction.

## 5. Conclusions

In summary, our study demonstrates that a higher plasma concentration of *myo*-inositol, particularly in early pregnancy, is associated with reduced blood loss post-delivery. This may indicate the potential for *myo*-inositol as a nutrition-based approach to be incorporated into routine care to reduce the risk of PPH and, consequently, further lower maternal morbidity and mortality globally.

## Figures and Tables

**Table 1:** Baseline characteristics of participants providing data for this study.

Characteristics		Total (N = 583)	
***Maternal***			
**Age** [years; mean (SD)]		30.3 (3.3)	
**Pre-pregnancy BMI** [kg/m^2^; median (IQR)]		23.7 (21.3–26.9)	
**Ethnicity**, n (%)			
White Caucasian		345 (59.2)	
Chinese		146 (25.0)	
South Asian (Indian, Pakistani, Bangladeshi)		30 (5.2)	
Malay		23 (3.9)	
Other (Mixed, Black, Polynesian)		39 (6.7)	
**Site**, n (%)			
UK		189 (32.4)	
SG		166 (28.5)	
NZ		228 (39.1)	
**Household income for country**, n (%)			
1st quintile (lowest)		7 (1.2)	
2nd quintile		43 (7.4)	
3rd quintile		124 (21.3)	
4th quintile		202 (34.7)	
5th quintile (highest)		184 (31.6)	
Unavailable		23 (3.8)	
**Preconception smoking**, n (%)			
Previous smoker		93 (16.1)	
Active smoker		23 (4.0)	
**Nulliparous**, n (%)		369 (63.3)	
**Previous cesarean** (denominator—all parous women), n (%)		61 (28.5)	
**Mode of delivery**, n (%)			
Vaginal delivery		414 (71.0)	
Cesarean section in labor		94 (16.1)	
Cesarean section without labor		75 (12.9)	
***Neonatal***			
**Gestational age at birth** [weeks; median (IQR)]		39.4 (38.5–40.3)	
**Birthweight** [kg; median (IQR)]		3.3 (3.0–3.7)	
**Size at birth ^1^**, n (%)			
LGA > 90th centile		43 (7.4)	
AGA		495 (84.9)	
SGA < 10th centile		45 (7.7)	
***Nutrient***	**Median concentration at** **pre-pregnancy baseline** (IQR)	**Low status at pre-pregnancy baseline,** n (%)	**Low status** **definition**
Vitamin B2 (nmol/L)	13.0 (8.0–20.9)	45 (7.7%)	<5
Vitamin B6 (nmol/L)	60.4 (43.7–97)	10 (1.7%)	<20
Vitamin B12 (pmol/L)	352.2 (276.1–435.5)	62 (10.6%)	<221
Vitamin D (nmol/L)	53.8 (40.2–68.9)	244 (41.9%)	<50
*Myo*-inositol (µmol/L)	21.9 (19.2–25.5)	NA	NA

^1^ The Royal College of Paediatrics and Child Health (RCPCH) growth charts [23]. Abbreviations: AGA, appropriate-for-gestational-age; BMI, body mass index; IQR, interquartile range; LGA, large-for-gestational-age; n, number; NA, not applicable; NZ, New Zealand; SD, standard deviation; SG; Singapore, SGA, small-for-gestational-age; UK, the United Kingdom.

## Data Availability

Individual participant data may be shared with an appropriately qualified individual working in an appropriate institution where an institutional signatory can confirm the recipient’s adherence to relevant information safeguards stipulated in a formal Data Transfer Agreement. Reasonable requests can be made through Professor Nicholas Harvey (nch@mrc.soton.ac.uk), as Director of the MRC Lifecourse Epidemiology Centre.

## Supplementary Materials

The following supporting information can be downloaded at: https://www.mdpi.com/article/10.3390/nu16132054/s1, Figure S1: participant flow chart; Figure S2: Associations of plasma concentrations of vitamins and *myo*-inositol with postpartum blood loss adjusting for site only.
