# Supplementary material for: Higher Plasma Myo-Inositol in Pregnancy Associated with Reduced Postpartum Blood Loss: Secondary Analyses of the NiPPeR Trial
Source: Nutrients. 2024 Jun 27;16(13):2054. doi: 10.3390/nu16132054 (PMC11242953; doi:10.3390/nu16132054)
Supplement: Supplementary file 1 [file nutrients-16-02054-s001.zip › nutrients-3076929-supplementary.pdf]

Supplementary material

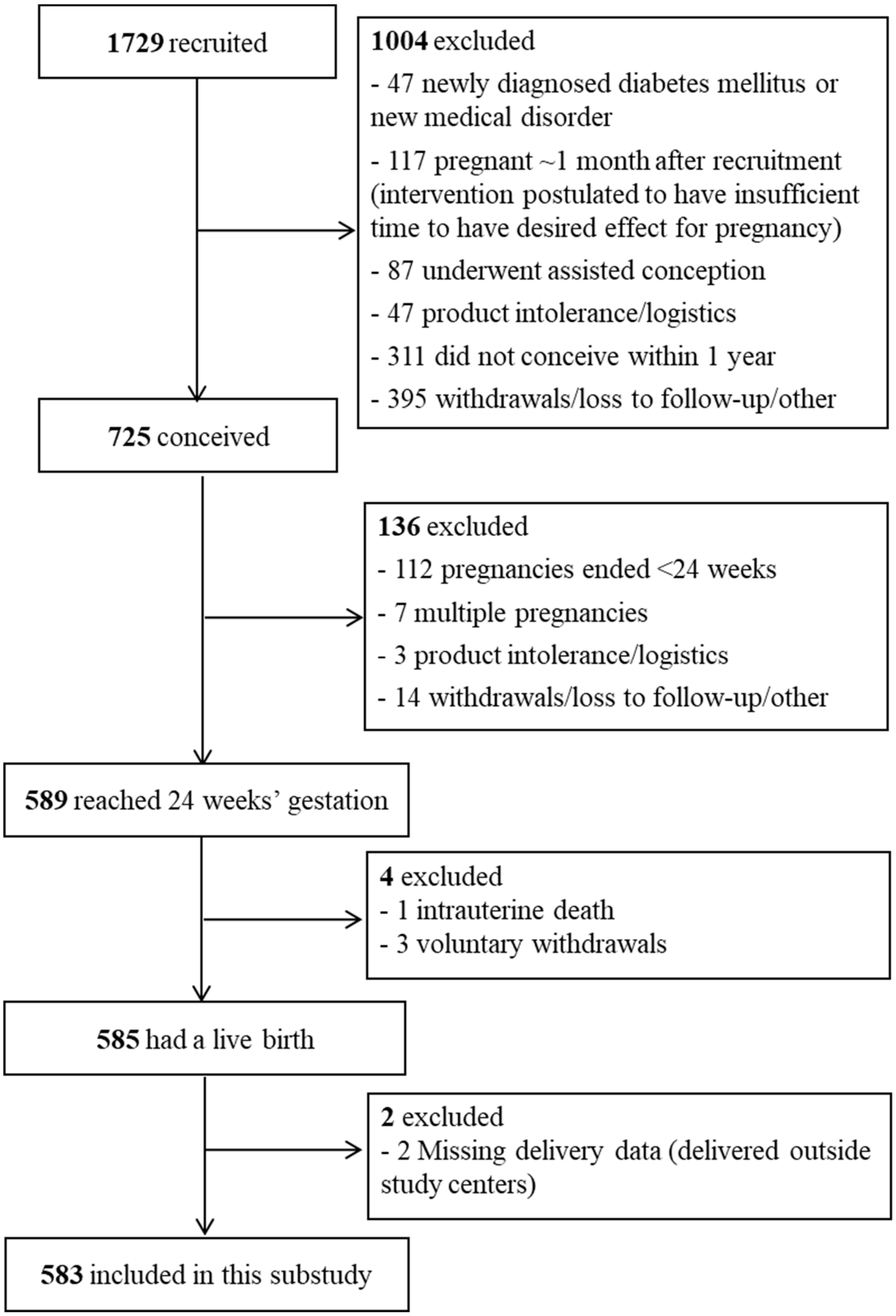

Supplementary Figure S1: Participant flow chart.

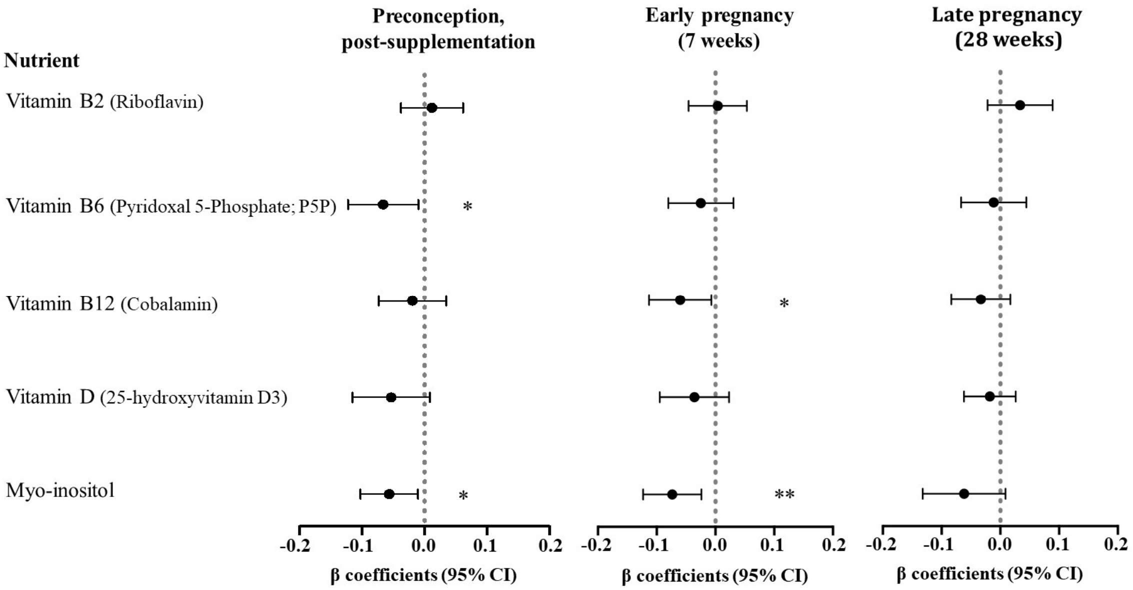

Supplementary Figure S2: Associations of plasma concentrations of vitamins and *myo*-inositol with postpartum blood loss adjusting for site only. Maternal plasma was collected at preconception post-supplementation (N=575), 7 weeks of gestation (N=558) and 28 weeks of gestation (N=575). Linear regression was utilized to analyze the associations of log_e-transformed and standardized plasma concentrations of vitamins and *myo*-inositol with log_e-transformed postpartum blood loss. Coefficients expressed as % change in ml blood loss per standard deviation (SD) increase in log_e nutrient concentration. Model fit (represented by R^2) for significant relationships: preconception vitamin B6 (0.111), 7-week vitamin B12 (0.117), preconception myo-inositol (0.112), and 7-week *myo*-inositol (0.123). Statistical significance: *p<0.05, **p<0.01. Abbreviations: CI, confidence interval.
